# The role of mitochondrial genes on nuclear gene expression in neovascular age related macular degeneration: analysis of nuclear VEGF gene expression after ranibizumab treatment in cytoplasmic hybrid retinal pigment epithelial cell lines correlated with clinical evolution

**DOI:** 10.1186/s40942-023-00476-7

**Published:** 2023-07-25

**Authors:** Rodrigo Donato Costa, Farid José Thomaz Neto, M. Tarek Moustafa, Shari R. Atilano, Marilyn Chwa, Javier Cáceres-del-Carpi, Mohamed Hamid Mohamed, M. Cristina Kenney, Baruch D. Kuppermann

**Affiliations:** 1 Gavin Herbert Eye Institute, University of California, 850 Health Sciences Road, Irvine, CA 92697 USA; 2 Instituto Donato Oftalmologia, Poços de Caldas, Brazil; 3 Ophthalmology Department, Minia University, Minia, Egypt; 4 Facultad de Medicina Hipólito Unanue, Universidad Nacional Federico Villareal, Lima, Perú

## Abstract

**Purpose:**

The present study tests the hypothesis that mitochondrial genes have retrograde signaling capacity that influences the expression of nuclear genes related to angiogenesis pathways. Cytoplasmic hybrid (cybrid) in vitro cell lines with patient specific mitochondria inserted into an immortalized retinal pigment epithelial cell line (ARPE-19) were used to test this hypothesis. This type of analysis can provide important information to identify the optimal regimen of anti-VEGF treatment, personalizing age-related macular degeneration (AMD) therapies.

**Methods:**

Mitochondria deficient ARPE-19 cells (Rho0) were fused with AMD donor’s platelets to create individual cybrid cell lines containing mitochondria from patients with phenotypic AMD disease and nuclear DNA from the immortalized RPE cell line. The cybrids were treated with Ranibizumab (Lucentis, Genentech, San Francisco, CA), at 4 different concentrations for 24 h, and subsequently the levels of reactive oxygen species (ROS), gene expression for *VEGF-A*, hypoxia-inducible factor 1-alpha (*HIF1-a*) and manganese superoxide dismutase (*SOD2)* were measured. The clinical evolution of the two AMD-donors were correlated with the molecular findings found in their ‘personalized’ cybrids.

**Results:**

Cybrids from Patient-01 showed down-regulation of gene expression of *VEGF-A* and *HIF-1a* at both 1X and 4X Ranibizumab concentrations. Patient-01 AMD cybrid cultures had an increase in the ROS levels at 1X (P = 0.0317), no changes at 2X (P = 0.8350) and a decrease at 4X (P = 0.0015) and 10X (P = 0.0011) of Ranibizumab. Clinically, Patient-01 responded to anti-VEGF therapy but eventually developed geographic atrophy. Patient-02 cybrids demonstrated up-regulation of gene expression of *VEGF-A* and *HIF-1a* at Ranibizumab 1X and 4X concentrations. There was decreased ROS levels with Ranibizumab 1X (P = 0.1606), 2X (P = 0.0388), 4X (P = 0.0010) and 10X (P =  < 0.0001). Clinically, Patient-02 presented with a neovascular lesion associated with a prominent production of intraretinal fluid in clinical follow-up requiring regular and repeated intravitreal injections of Ranibizumab with recurrent subretinal fluid.

**Conclusions:**

Our cybrid model has the potential to help personalize the treatment regimen with anti-VEGF drugs in patients with neovascular AMD. Further investigation is needed to better understand the role that the mitochondria play in the cellular response to anti-VEGF drugs. Future studies that focus on this model have the potential to help personalize anti-VEGF treatment.

## Introduction

Age-related macular degeneration (AMD) is the leading cause of vision loss in the elderly population in developed countries worldwide. In the United States, 1.5% of the population will progress to blindness because of AMD. In the adult population over 80 years old, 11.8% lose vision due to advanced stages of macular degeneration [1–3]. The disease usually develops in three stages: Early AMD—mild retinal pigment epithelium (RPE) abnormalities are associated with scattered medium-sized drusen. Intermediate AMD—characterized by mild extra-macular geographic atrophy, several medium-sized drusen (≥ 63 μm but < 125 μm) and at least one large drusen (≥ 125 μm) (30). There are two forms of advanced AMD: the first is the Dry or Atrophic form that has extensive geographic atrophy in the central macula area, and the second form is the Wet, exudative or neovascular form with a neovascular complex beneath the macular area. The advanced forms of AMD (dry and wet) are correlated with most extensive visual loss [2].

In the last two decades, the exudative form of AMD has been treated with anti-vascular endothelial growth factor (anti-VEGF) drugs, which decreases neovascularization and exudation, and improves the patient’s vision and quality of life. However, there is often a subset of patients that does not show significant improvement and demonstrates clinical differences in the responses to the three historically available anti-VEGF drugs: Ranibizumab (Lucentis^®^; Genentech, San Francisco, California, USA), Aflibercept (Eylea^®^; Regeneron, Tarrytown, New York, USA) and Bevacizumab (Avastin^®^; Genentech, San Francisco, California, USA). Ranibizumab and Aflibercept are approved by the U.S. Food and Drug Administration (FDA) for the treatment of wet AMD, Bevacizumab is approved by the FDA for treatment of different types of cancer in different organs. Currently, Bevacizumab off-label use in ophthalmology is primarily supported by the Comparisons of Age-Related Macular Degeneration Treatments Trial (CATT) study that proved comparable efficacy of Bevacizumab in the treatment of AMD compared with Ranibizumab [4–8], though many other subsequent studies have shown comparable benefits of bevacizumab for the treatment of neovascular AMD. [9].

In 2006, Rosenfeld and colleagues presented the results of the MARINA and ANCHOR studies showing the efficacy of monthly Ranibizumab intravitreal injections [10]. Although the monthly injections are the “gold standard” treatment, it is difficult for the patients and expensive. Studies such as PIER, PrONTO, and SAILOR have documented decreased retina thickening by optical coherency tomography (OCT) and improved final visual acuity when Pro Re Nata (PRN) or Treat-and-Extend algorithms have been used as the alternative regimens, [10–15], but so far there is no unanimous decision by the clinicians.

Mitochondria are one of the most important organelles for cellular function and survival, providing chemical energy through ATP production, controlling the cellular metabolism and in the regulation of programmed cell death (apoptosis). The retinal pigment epithelial cells (RPE) have a high density of mitochondria and robust metabolic activity [16]. Mitochondria, which contain maternally inherited, circular mitochondrial DNA (mtDNA) have been implicated in the development and progression of AMD. As shown by Liang and Godley [17], mtDNA damage is probably more relevant to the mitochondrial theory of aging than damage to protein or lipids. Many authors have shown that chronic mitochondrial dysfunction is associated with AMD and others degenerative diseases [17–20]. In AMD, aging and oxidative stress are important factors in the pathogenesis. It is possible to create cytoplasmic hybrid (cybrid) cell lines using immortalized retinal cell lines that lack their own mtDNA (Rho*0* cells) and inserting mitochondria from well-characterized AMD patients. These ‘personalized’ cybrid cell lines have identical nuclei but mitochondria from different individuals so if the cybrid cell lines have different molecular/biochemical profiles it is most likely due to the modulation effects of the individual’s mitochondria. This allows the ability to study the role of ‘personalized’ phenotypic disease state mitochondria in influencing nuclear gene expression, in order to better understand variances in response to therapeutic regimens.

Reactive oxygen species (ROS) are free radicals, strong oxidizing agents or oxygen species elevated to a higher energy level in the cells. They are highly reactive molecules which can cause oxidative damage to lipids, proteins, and nucleic acids. Mitochondria are a major endogenous source of elevated levels of ROS, which can be correlated with mtDNA damage [16–18]. This study was designed to identify the best regimen of anti-VEGF treatment for each patient by correlating their in vitro gene expression to their clinical response to anti-VEGF treatment.

## Materials and methods

### Cybrid culture

The AMD donors’ blood was collected via venipuncture in tubes containing 3.2% sodium citrate and platelets were isolated by a series of centrifugation steps. Final pellets were suspended in tris buffered saline. ARPE-19 cells are a cell line derived from human retinal epithelial cells (ATCC, Manassas, VA, USA). These cells show structural and functional properties similar to human retinal pigmented epithelium (RPE) and are commonly used in retinal research [21, 22]. The ARPE-19 cells were made deficient in mitochondria (Rho*0*) by serial passages in low dose ethidium bromide. Polyethylene glycol was used to fuse the platelets with Rho*0* ARPE-19 cells to create the personalized cybrid cells, according to modified procedures of Chomyn [23]. We verified the transfer of the mitochondria into the Rho*0* ARPE-19 cells by using polymerase chain reaction (PCR), restriction enzyme digestion and sequencing the mtDNA [24]. In the cybrid cell lines, the nuclei of all cells were identical, but each cell line had mitochondria from different AMD individuals (either Patient-01, Cybrid 14–141 or Patient-02, Cybrid 14–144). Therefore, if the cell lines behaved differently, it is likely due to the influence of the patient specific mitochondria. Cybrids were cultured in DMEM-F12 containing 10% dialyzed fetal bovine serum, 17.5 mm glucose, 100 unit/ml penicillin, 100 $\mu g/ml$ streptomycin, 2.5 $\mu g/ml$ fungizone and 50 $\mu g/ml$ gentamycin until they became confluent.

The mtDNA haplogroup of each patient and their corresponding cybrid cell line was identified and confirmed by sequencing the mtDNA. Since the mtDNA haplogroup patterns can modulate the cybrid cell’s behavior and response to drugs (32–33), these experiments selected only subjects with the H mtDNA haplogroup.

### Reactive oxygen species (ROS) assay

The cybrids containing mitochondria from either AMD subjects (AMD) were treated with Ranibizumab at 4 different concentrations and then the levels of ROS were measured as described by Atilano et al. [25]. Briefly, cybrids were cultured in 24-wells plates for 48 h and then treated with Ranibizumab in 1X (one-time clinical intravitreal dose by concentration assuming a 4 ml vitreous volume), 2X (two-times clinical dose), 4X (4-times clinical dose), and 10X (10-times clinical dose) concentrations of the human clinical dose. The human clinical dose of Ranibizumab (X) was calculated by assuming that the amount of 0.5 mg/ 0.05 ml of Ranibizumab is distributed equally throughout the 4 ml of human vitreous after being injected into the human eye. After 24 h exposure to Ranibizumab, ROS production levels were measured in the cybrids using the fluorescent dye 2’7’-dichlodihydrofluorescein diacetate reagent (H_2_DCFDA, Invitrogen—Molecular Probes, Carlsbad, CA, USA.) and fluorescent plate reader (Spectramax Gemini XPS—Molecular Devices, Sunnyvale, CA, USA.). Results were normalized to untreated cells. All the experiments were performed in quadruplicate and repeated at least three times.

### Isolation of RNA and synthesis of cDNA

Cells from cybrid cultures (age-matched cybrids, n = 2 and AMD cybrids, n = 2) were pelleted and RNA isolated using the PureLink RNA Mini kit (ThermoFisher Scientific, Waltham, MA) following the manufacturer’s protocol. The RNA was quantified using a NanoDrop1000 (ThermoScientific). For qRT-PCR analyses, 2ug of individual RNA samples were reverse transcribed into cDNA using the SuperScript VILO IV Master Mix (ThermoFisher Scientific) [26].

### Quantitative reverse transcriptase PCR (qRT-PCR) analyses

The qRT-PCR analyses were performed on the cDNA of individual age-matched cybrids (n = 2) and AMD cybrids (n = 2) and not on pooled samples using Power SYBR on the Viia7 Real-Time PCR System (ThermoFisher Scientific). The gene expression levels were standardized for all primers (Table 1) using hypoxanthine phosphoribosyltransferase 1 (HPRT1) as the reference gene. All analyses were performed in triplicate. The fold values were calculated using the 2^(-∆∆Ct) formula.

**Table 1:** Description of qRT-PCR Genes

Symbol	Gene Name	RefSeq Number	Function
HIF1A	hypoxia inducible factor 1 subunit alpha	NM_001530	This gene encodes the α subunit of transcription factor hypoxia-inducible factor-1 (HIF-1), HIF-1 functions as a master regulator of cellular and systemic homeostatic response to hypoxia by activating transcription of many genes, including those involved in energy metabolism, angiogenesis, apoptosis, and other genes whose protein products increase oxygen delivery or facilitate metabolic adaptation to hypoxia
SOD2	superoxide dismutase 2	NM_000636	A member of the iron/manganese superoxide dismutase family, this gene encodes a mitochondrial protein that forms a homotetramer and binds one manganese ion per subunit. This protein binds to the superoxide byproducts of oxidative phosphorylation and converts them to hydrogen peroxide and diatomic oxygen
VEGFA	Vascular endothelial growth factor A	NM_003376	A member of the PDGF/VEGF growth factor family, this gene encodes a heparin-binding protein, which exists as a disulfide-linked homodimer. This growth factor induces proliferation and migration of vascular endothelial cells, and is essential for both physiological and pathological angiogenesis
HPRT1	Hypoxanthine phosphoribosyltransferase 1	NM_000194	Transferase which catalyzes conversion of hypoxanthine to inosine monophosphate and guanine to guanosine monophosphate. Critical to generation of purine nucleotides through the purine salvage pathway. Endogenous control for QPCR

Statistical analyses of gene expression levels were performed to measure difference between the age-matched and AMD cybrids individually using Prism, version 5.0 (GraphPad Software Inc., San Diego, CA).

## Results

### Clinical history for patient-01 (CYBRID 14–141) and patiet-02 (CYBRID 14–144)

#### First case—clinical history of patient 01 (CYBRID 14–141)

**Fig. 1:**
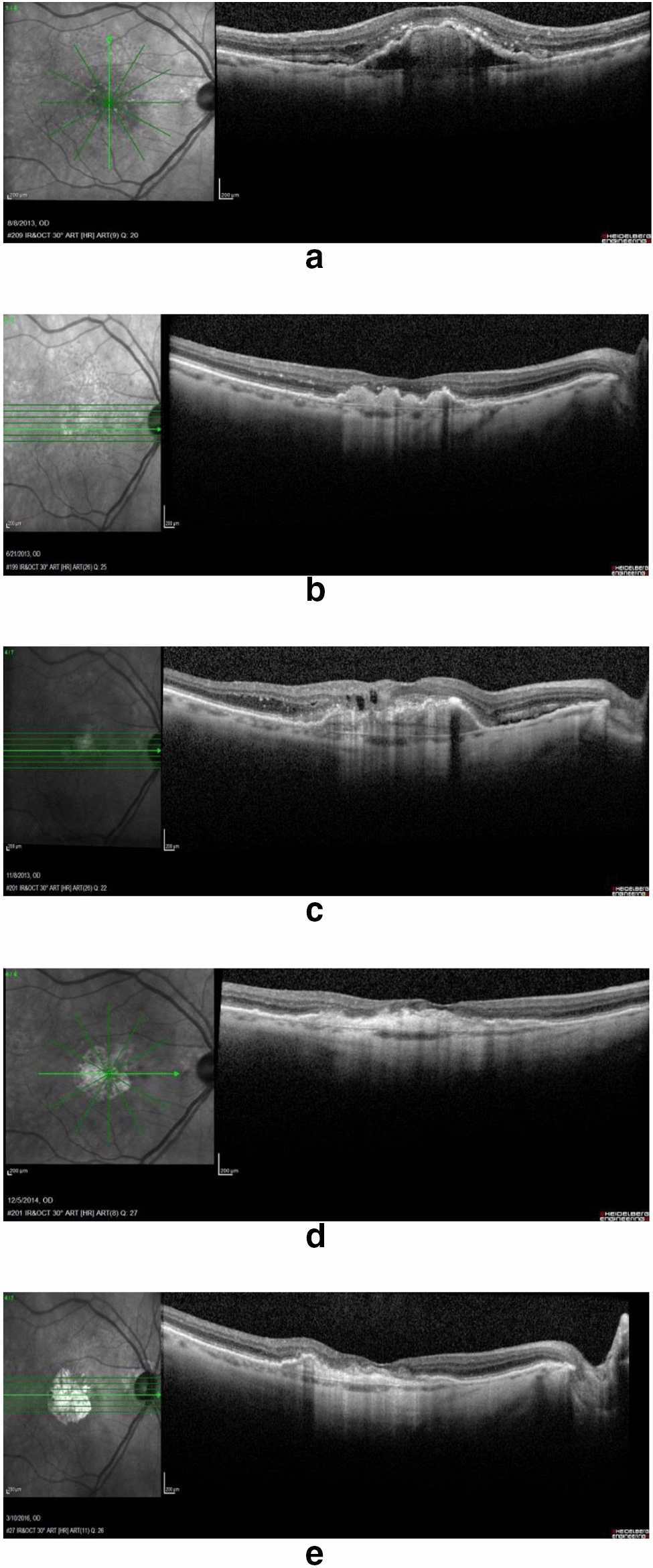
Case report for patient-01 (CYBRID 14–141). Clinical history of blood donor of cybrid 14–141**: a**. Right Eye before treatment. **b**. Right Eye after 3 intravitreal injections of Ranibizumab. **c.** Right Eye after 4 intravitreal injections of Ranibizumab. **d**. Right Eye after 8 intravitreal injections of Ranibizumab. **e.** Right Eye after 8 intravitreal injections of Ranibizumab + 4 intravitreal injections of Aflibercept. No more injections in the last months, lesion remains dry in right eye

The first cybrid cell line (Cybrid line 14–141) was created from an 80-year-old Caucasian, female donor that presented with initial right eye visual acuity of 20/40 due to wet AMD. The fundus exam showed diffuse reticular pseudodrusen and cuticular drusen within the posterior pole (Fig. 1a). Optical Coherence tomography (OCT) images then were acquired using the Spectralis (Heidelberg Engineering, Heidelberg, Germany) (Fig. 1a–e).

**Fig. 2:**
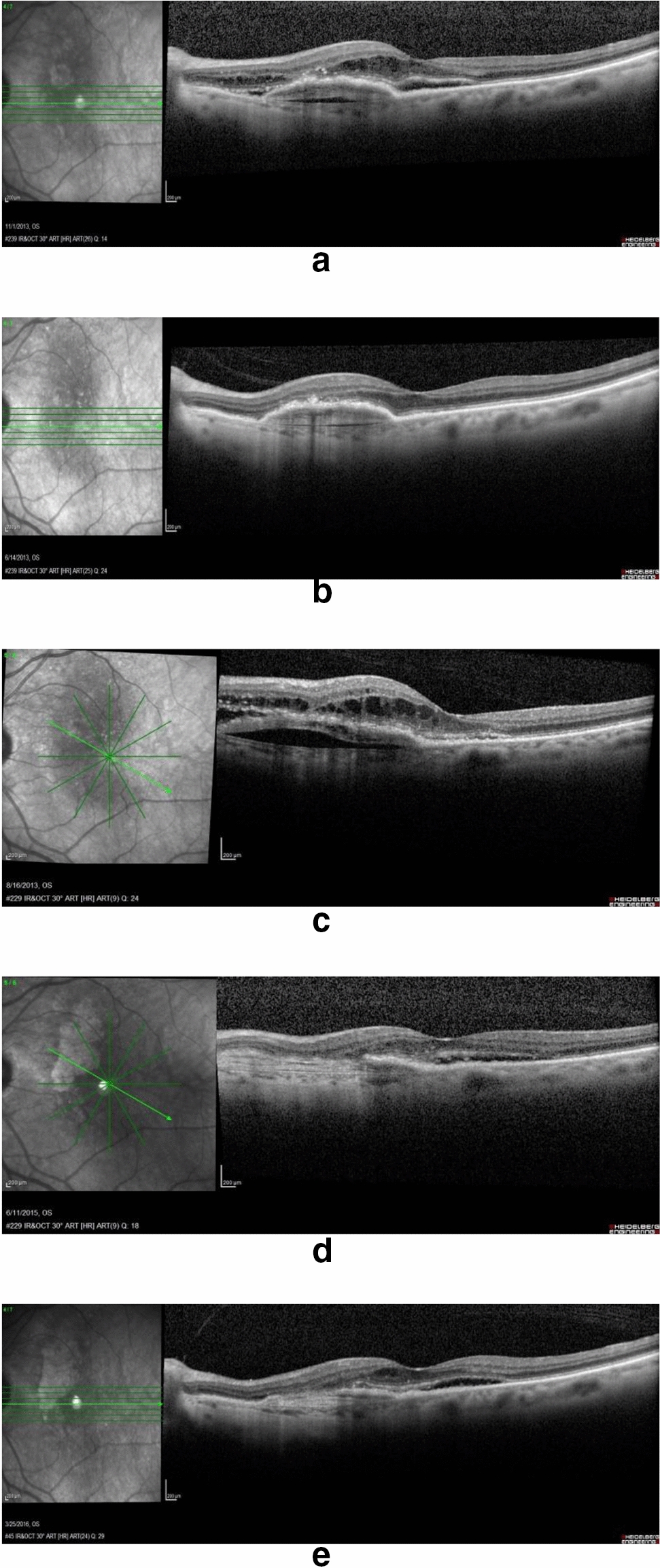
**a.** Left Eye before treatment. **b.** Left Eye after the 3rd intravitreal injection of Ranibizumab. **c.** Left Eye after the 4th intravitreal injection of Ranibizumab. **d.** Left Eye after the 12th intravitreal injection of Ranibizumab. **e.** Left Eye after the 18th intravitreal injection of Ranibizumab. Lesion remains producing fluid in the left eye

Initially, Patient-01 received intravitreal injections of Ranibizumab (Lucentis^®^), and was followed every 4–6 weeks using a PRN regimen. Over a two-year period, the Patient-01 received a total of eight [8] intravitreal Lucentis^®^ injections (IVL). After two years, the treatment was switched to Aflibercept (Eylea^®^) and over the subsequent year, Patient-01 received a total of four [4] intravitreal Eylea injections (IVE). The patient was followed for another year but no other injections were needed. During these four years, the subretinal neovascular membrane progressed to a disciform scar lesion associated with geographic atrophy (Fig. 2).

Upon reviewing the chart and OCT images, clinically it was found that Patient-01 had a good response with Ranibizumab (IVL) treatments, which was effective in almost completely eliminating the intraretinal and subretinal fluids. At visit #2 (PRN regimen), the Patient-01 presented with a dry macula. At visit #3, (4–6 weeks) and the visit #4, the macula was dry, therefore skipping an IVL injection. When the OCT had mild intraretinal fluid during the following visits a new Ranibizumab injection was done. The blood was donated to create the cybrid after 8 injections of Ranibizumab (IVL) in the right eye.

#### Second case patient-02 (CYBRID 14–144)

The second cybrid cell line analyzed (cybrid 14–144) was created from Patient-02, an 84-year-old Caucasian, female donor that presented with decreased vision in the left eye (20/150) due to wet AMD. The initial exam showed the presence of subretinal neovascular membrane in posterior pole. The Optical Coherence tomography (OCT) images were acquired using the Spectralis (Heidelberg Engineering, Heidelberg, Germany). In the exam, subretinal hyper-reflective material (subretinal neovascular membrane) associated with prominent subretinal fluid, few intra-retinal fluidic cysts, and retinal epithelial changes in posterior pole were observed. Initial visual acuity in the left eye was 20/150.

Patient-02 began to receive anti-VEGF treatment using Ranibizumab (Lucentis^®^ – IVL). Patient-02 was followed every 4 to 6 weeks and treated following pro re nata (PRN) regimen. Over a 2.5 year period, the patient received a total of eighteen [18] intravitreal Lucentis injections (IVL) due to recurrent subretinal fluid, requiring recurrent injections every 8 weeks.

Patient-02 donated a blood sample in order for cybrids to be created (Cybrid 14–144) after the patient had received 9 intravitreal injections of Ranibizumab to the left eye for the recurrent fluid accumulation.

### Biochemical and molecular analyses for patient-01 (CYBRID 14–141) and patient-02 (CYBRID 14–144)

#### Reactive oxygen species (ROS) in AMD donors cybrids

**Fig. 3:**
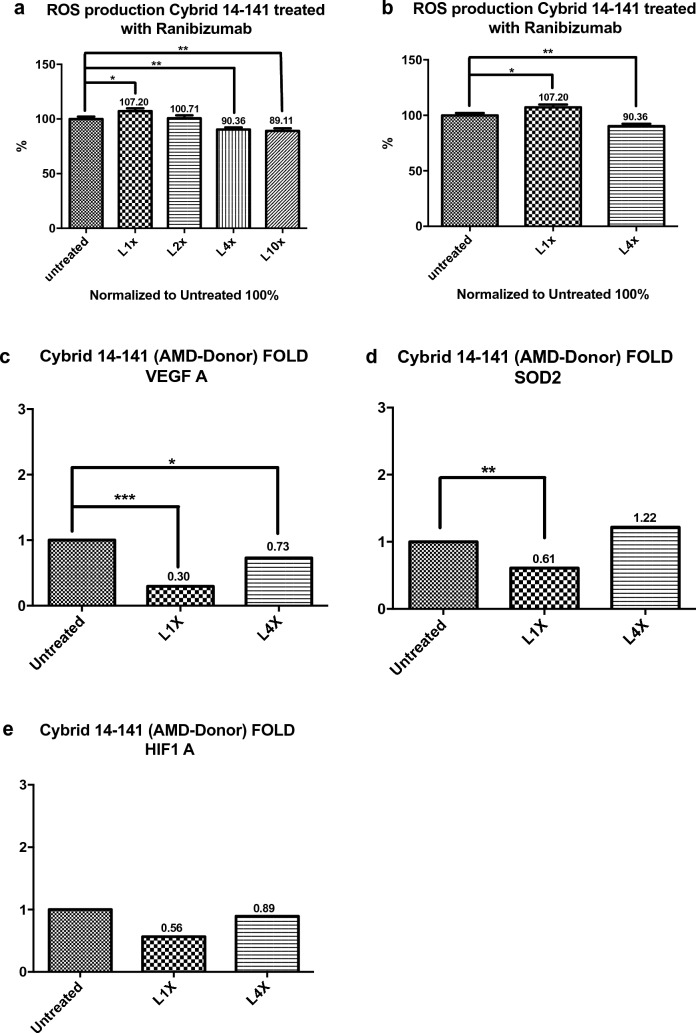
**a**–**e** Cybrid 14–141 ROS levels at 1X, 2X, 4X, 10X and untreated. Fold values of *VEGF-A, HIF1-A, and SOD2* gene expression after treatment at 1X, 4X and untreated

**Fig. 4:**
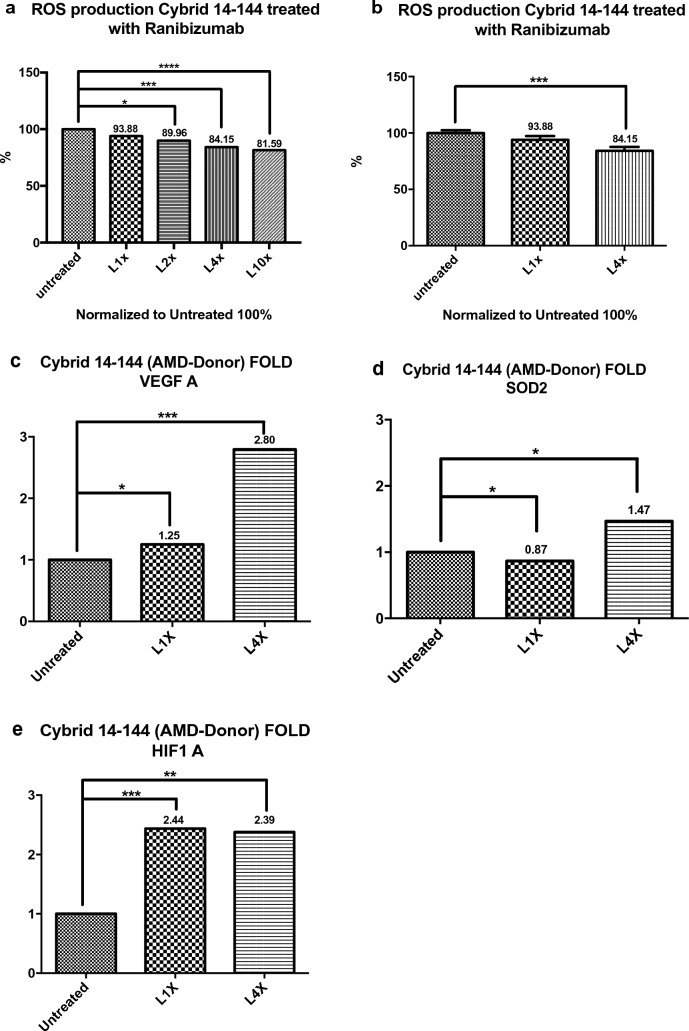
**a**–**e** Cybrid 14–144 ROS levels at 1X, 2X, 4X, 10X and untreated. Fold values of *VEGF-A, HIF1-A, and SOD2* gene expression after treatment at 1X, 4X and untreated

The two cybrids treated with Ranibizumab showed differing results on ROS assay. Cybrid 14–141 showed an increase in the ROS levels at 1X (P = 0.0317), no changes at 2X (P = 0.8350) and a decrease at 4X (P = 0.0015) and 10X (P = 0.0011) the human clinical dose concentration of Ranibizumab (Fig. 3a, b). Cybrid 14–144 showed a decrease in the ROS levels at 1X (P = 0.1606, not statistically significantly) and a decrease at Ranibizumab 2X (P = 0.0388), 4X (P = 0.0010) and 10X (P =  < 0.0001) compared to the untreated control cultures (Fig. 4a, b).

After the ROS results were analyzed, it was decided to continue working with the human clinical dose concentration (1X) and the higher clinical dose concentration of Ranibizumab used in publications (4X—4 times the usual dose, 2.0 mg/0.05 mL) [29].

#### Quantitative-real-time PCR (qRT-PCR) analysis in AMD donor cybrids

The two cybrids treated with Ranibizumab showed different results of gene expression levels. Cybrid 14–141 showed a statistically significant reduction in expression of *VEGF-A* after treatment with Ranibizumab at 1X (P =  < 0.0001) and 4X (P = 0.0313) when compared to untreated cells. A reduced expression of the *HIF1-A* gene at 1X (P = 0.0608) and 4X (P = 0.6365) when compared to untreated cells was also seen. A statistically significant decrease in the expression of SOD2 at 1X (P = 0.0083) when compared to untreated cells was seen and an elevated but not statistically significantly expression level at 4X (P = 0.0615) when compared to untreated cells was seen (Fig. 3c–e). Contrastingly, Cybrid 14–144 showed an elevated expression of *VEGF-A* gene after treatment with Ranibizumab at 1X (P = 0.0146) and 4X (P =  < 0.0001) when compared to untreated cells. A statistically significant elevated expression of *HIF1-A* at 1X (P = 0.0002) and 4X (P = 0.0011) was seen when compared to untreated cells. Also, a reduced expression of *SOD2* at 1X (P = 0.0208) and an elevated expression at 4X (P = 0.0314) when compared to untreated cells was found (Fig. 4c–e).

## Discussion

Since the beginning of the anti-VEGF treatment era in 2005 and multiple subsequent clinical studies, the management of AMD has had a profound evolution with the introduction of new drugs. However, even with all the developments, there is still debate on how to determine the best regimen of anti-VEGF drugs injections for an individual patient.

Most physicians have settled on two major regimens to delivery anti-VEGF drugs: (a) Pro Re Nata (PRN) in which the patient is evaluated every 4 to 6 weeks and the decision to proceed or not with the anti-VEGF injection is based on the clinical exam and OCT findings, or (b) Treat-and-Extend basis, where upon the physician starts the treatment and progressively increases the period between injections. [11–13].

The gold standard treatment of monthly anti-VEGF injections is costly and often difficult for the patient to be followed. In addition, this treatment may be excessive as some patients progress to geographic atrophy in the posterior pole and macular area, perhaps because VEGF proteins are associated with cell survival [4–7] and repetitive anti-VEGF injections may reduce the VEGF levels required for cell health.

The ideal goal for all physicians working with Exudative Age-related Macular Degeneration (wAMD) patients would be ‘*personalized treatment*’ with anti-VEGF drugs. To move towards this goal, we have developed the AMD cybrid model, which are cell lines with identical nuclear genome but mitochondria from different wAMD donor subjects. Our previous studies have demonstrated that cybrids with AMD mitochondria have accelerated cell death and increased expression of RNA/proteins in pathways related to apoptosis, inflammation, and autophagy [27]. We propose that the cybrids are a reliable model to test the response patterns to different drugs and correlate the in vitro responses to the clinical patterns found in the AMD patients. Using the cybrid model treated with Ranibizumab in different concentrations, it was possible to correlate the changes found in gene expression of *VEGF-A* and *HIF1-A* with the clinical evolution of these two patients that were treated clinically with IVL for wAMD.

The clinical history of these two patients had relative similarity in treatment duration and medications used (Ranibizumab (Lucentis^®^). Clinically, the patients were female, in their 80’s and both with the same mtDNA H haplogroup profile. While the patients had a good evolution using Ranibizumab injections, the behavior of the lesion’s evolution was not the same.

After 8–9 IVL injections, both patients donated their blood to create the cybrids. The cybrid created from the blood of the Patient-01 (Cybrid 14–141) demonstrated a down-regulation of gene expression of *VEGF-A* and *HIF1-A*, 24 h after treatment with Ranibizumab at 1X and 4X concentrations in vitro. Clinically while using IVL, Patient-01 presented with fluid in some visits and was dry at other visits. After injection #8 of Ranibizumab, he was switched and received 4 additional injections of Aflibercept. The lesion remained dry for more than 14 months and eventually progressed to a disciform scar with geographic atrophy.

The cybrid created from Patient-02 (Cybrid14-144) demonstrated significant up-regulation of *VEGF-A* and *HIF1-A* in cultures treated 24 h with Ranibizumab at 1X and 4X concentrations. Clinically, in the clinical follow-up (PRN), Patient-02 showed a neovascular lesion associated with prominent production of intraretinal fluid. After each injection the lesion was dry initially but developed subretinal fluid by the follow-up visit. Patient-02 received 18 IVL between February 2013 and October 2016.

Analyzing these two patients, it becomes apparent that in response to Ranibizumab treatment in vitro, the Cybrid 14–141 cell line for Patient-01 showed down-regulation of the expression of *VEGF-A* and *HIF1-A* genes. In this case, the cybrid model paralleled the clinical situation. The in vitro Cybrid 14–141 model showed a positive response to the Ranibizumab treatment (lower *VEGF-A* and *HIF1-A* levels), that matched the in vivo clinical picture of reduced fluid accumulation after the IVL injections were made. Based on the in vitro cybrid model and in vivo clinical picture, one can speculate that is better to follow the Pro Re Nata (PRN) regimen for a patient exhibiting this behavior, only making anti-VEGF injections when OCT and examination show a reactivation of the lesion and retinal fluid production. This approach may ultimately protect the retina and perhaps avoid development of geographic atrophy.

In contrast, the Cybrid 14–144 cell line for Patient-02 showed up-regulation of the pro-angiogenic genes, *VEGF-A* and *HIF1-A*, after treatment with Ranibizumab (1 × and 4 × concentrations). The in vitro Cybrid 14–144 model correlated with the clinical picture for Patient-02, who showed prominent subretinal fluid and intraretinal cysts despite regular IVL of Ranibizumab treatments. These results support that Patient-02 would benefit from the Treat-and-Extend regimen, making regular injections without waiting for the reactivation of lesion and the retinal fluid production. This approach has a potential to improve the retina anatomy and the final visual acuity of this patient.

It should be noted that these different cybrid cell lines (Cybrid 14–141 or Cybrid 14–144) have the same nuclei but mitochondria from different AMD patients (Patient-01 or Patient-02) [28]. The strong correlation found between AMD cybrid gene expression after Ranibizumab treatment and the clinical history of these two patients shows that the AMD mitochondria likely have retrograde signaling capacity to modulate expression of multiples genes related to angiogenesis.

**Fig. 5:**
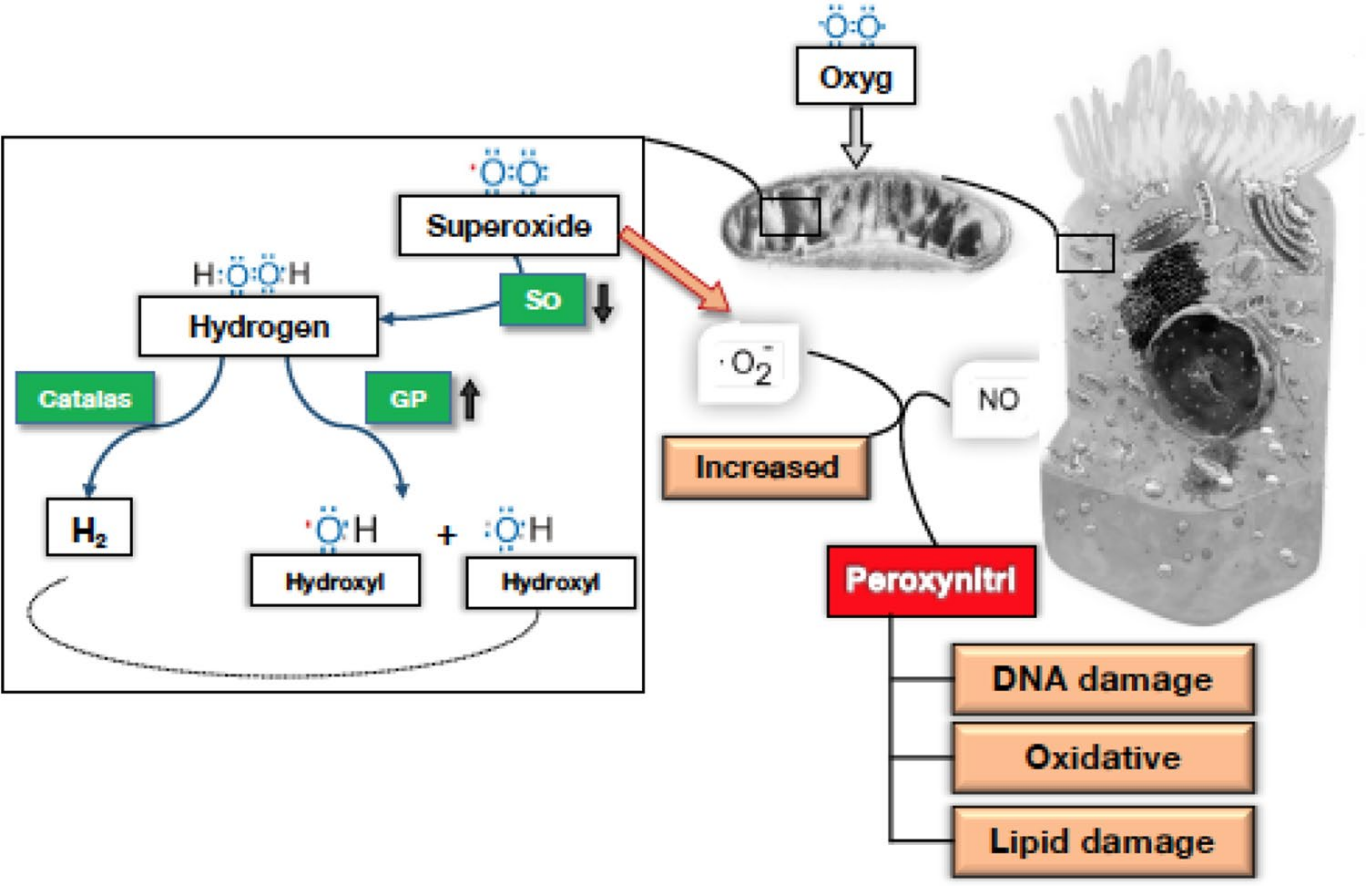
Correlation between SOD2 and Cellular Superoxide. SO, superoxides; GP, glutathione peroxidase; Catalas, catalase; Peroxynitrl, peroxynitrile; Oxyg, oxygen; NO, nitric oxide; O^−^_2_, singlet oxygen, superoxide

SOD2 is localized within the mitochondria and responsible for eliminating the harmful reactive oxygen ions (O^−^_2_) before extensive damage occurs. During oxidative phosphorylation (OXPHOS), these superoxides are produced from reactions associated with complexes I and III. The SOD2 enzyme rapidly converts the superoxide to hydrogen peroxide and diatomic oxygen. Down-regulation of SOD2 leads to higher cellular oxidative stress and ROS levels by increasing the free superoxide in the cell (Fig. 5). An up-regulation of SOD2 will remove free cellular superoxides, thereby decreasing the cellular oxidative stress and lowering ROS levels, which was observed in 4X-treated Cybrid 14–144 cultures that showed a 0.47-fold increase of SOD2 expression (P < 0.05) and a decrease in the ROS production level (P = 0.001). These data support that Ranibizumab may reduce oxidative stress by upregulation of antioxidant enzyme, SOD2, which would be beneficial for the retinal cells.

## Conclusion

Since AMD cybrids have identical nuclei, the different responses to Ranibizumab (Lucentis^®^) are likely due to the presence of mitochondria from different AMD patients. We propose that AMD cybrids may be a useful tool to gain information about how a wAMD patient may respond to anti-VEGF treatment, thereby guiding the clinician to more rapid, efficient treatment protocols. In the ‘*personalized*’ AMD cybrids, measuring the different expression levels of *VEGF-A* and *HIF1-A* genes in response to Ranibizumab have the potential to identify the best regimen of treatment with anti-VEGF. Further investigation is needed to better understand the role that the mitochondria play in the VEGF retrograde signaling after anti-VEGF treatment. Future studies should investigative additional patients to confirm the correlation between in vitro AMD cybrid responses to Ranibizumab and clinical responses of wAMD patients to antiVEGF treatments over monthly anti-VEGF treatments.

## Data Availability

The datasets used and/or analysed during the current study are available from the corresponding author on reasonable request.
